# Enhanced Antiviral Ability by a Combination of Zidovudine and Short Hairpin RNA Targeting Avian Leukosis Virus

**DOI:** 10.3389/fmicb.2021.808982

**Published:** 2022-02-16

**Authors:** Qun Wang, Qi Su, Bowen Liu, Yan Li, Wanli Sun, Yanxue Liu, Ruyu Xue, Shuang Chang, Yixin Wang, Peng Zhao

**Affiliations:** ^1^College of Animal Science and Veterinary Medicine, Shandong Agricultural University, Tai’an, China; ^2^Shandong Provincial Key Laboratory of Animal Biotechnology and Disease Control and Prevention, Tai’an, China; ^3^Shandong Provincial Engineering Technology Research Center of Animal Disease Control and Prevention, Tai’an, China

**Keywords:** avian leukosis virus, reverse transcriptase, zidovudine, RNAi, shRNA

## Abstract

Avian leukosis virus (ALV) causes tumor diseases in poultry and is circulating all over the world, leading to significant economic losses. In addition, mixed infection of ALV with other viruses is very common and is often reported to contaminate live vaccines. At present, there is no effective method to suppress the replication of ALV *in vitro*, so it is very difficult to remove it in mixed infection. As a retrovirus, the replication of ALV can be limited by reverse transcriptase (RT) inhibitors like zidovudine (AZT), but it also causes nontargeted cytotoxicity. To find the optimal solution in cytotoxicity and inhibition efficiency *in vitro* culture system, we firstly designed a combination therapy of AZT and short hairpin RNA (shRNA) targeting ALV and then verified its efficiency by multiple biological methods. Results showed that shRNA can effectively inhibit the expression of RT and then limit the replication of ALV. The combination of AZT and shRNA can significantly improve the antiviral efficiency in viral replication, shedding, and provirus assembly under the condition of low cytotoxicity. Overall, in this study, the combination therapy of AZT and shRNA targeting ALV showed excellent antiviral performance against ALV *in vitro* culture system. This method can be applied to multiple scenarios, such as the removal of ALV in mixed infection or the purification of contaminated vaccine strains.

## Introduction

Avian leukosis (AL) is an important oncogenic disease caused by avian leukosis virus (ALV; Payne and Nair, 2012). As an immunosuppressive virus, ALV is also easy to cause immune failure and secondary infection, resulting in huge economic losses (Su et al., 2019; Wang et al., 2020b). ALVs can be divided into 11 subgroups (designated A to K; Payne et al., 1992; Wang et al., 2012). Among them, ALV subgroup J (ALV-J) is the most pathogenic and epidemic subgroup, which is also highly prevalent in China (Gao et al., 2012; Lin et al., 2016; Su et al., 2018b; Zhou et al., 2019; Wang et al., 2020a). In addition, the long-term epidemic has also bred many recombinant or mutant strains and caused extensive mixed infection with other viruses (Cai et al., 2013; Su et al., 2018a,^2020^; Liang et al., 2019; Zhang et al., 2020). Recently, a lot of studies reported the ALV contamination in live vaccines and even the seed viruses (Mao et al., 2020). However, there is no effective method to suppress the replication of ALV *in vitro*, so it is very difficult to remove it in mixed infection.

Avian leukosis virus belongs to alpha retrovirus and has a positive-strand RNA genome (Nair and Fadly, 2013). The genomic structure of ALV is highly similar to that of the human immunodeficiency virus (HIV), whereas both of them rely on reverse transcriptase (RT) for genome replication (Hurwitz and Leis, 1972; Battula and Loeb, 1976; Nasioulas et al., 1995; Coffin and Fan, 2016). RT inhibitors like lamivudine (LAM) and zidovudine (AZT) were designed to inhibit the replication efficiency of retroviruses, and many of them have been proved to be effective in several viruses, especially the HIV (Durand-Gasselin et al., 2008; Akanbi et al., 2012; Clark and Hu, 2015; Berruti et al., 2021). However, the wide application of RT inhibitors has also led to the emergence of many drug-resistant virus strains, which may even cause a more serious epidemic (Fanning et al., 2003; Kantor et al., 2004; Bulteel et al., 2014; Mulato et al., 2021).

RNA interference (RNAi) is a process of silencing gene expression in a specific way mediated by miRNA or short hairpin RNA (shRNA), which gradually become a promising therapeutic approach for treating viral diseases (van Rij and Andino, 2006; DeVincenzo, 2008; Wang et al., 2017; Berkhout, 2018). RNAi targeting retrovirus mRNA has been verified to effectively inhibit the replication of human and animal retroviruses such as HIV (Bennasser et al., 2007; Scarborough and Gatignol, 2017), hepatitis B virus (Chen et al., 2003; Hamasaki et al., 2003), feline leukemia virus (Lehmann et al., 2015), and simian immunodeficiency virus (Lim et al., 2008).

Our previous work has confirmed that RT inhibitors also work efficiently in ALV and can be used to eradicate the contamination of ALV and other retroviruses in vaccine virus seeds (Wang et al., 2015; Cui et al., 2019). However, it is notable that, with the increase of drug concentration, the cytotoxicity is also increasing, which limits the further application of AZT. In the present study, we combined RNAi and RT inhibitors to achieve higher inhibition of virus replication with low cytotoxicity, and we aimed to establish an effective method to remove ALV in mixed infection or contaminated vaccine seed viruses *in vitro* culture system.

## Materials and Methods

### Cells, Virus, and Drugs

Avian leukosis virus subgroup J strain SDAU1005 (GenBank, access no. KT156668) was isolated from crossbreed broilers with fibrosarcoma, and the infectious clone and rescued virus of SDAU1005 was constructed and preserved in our laboratory (Wang et al., 2016). DF-1 cells were purchased from ATCC (United States) and cultured in Dulbecco’s Modified Eagle Medium (DMEM; Gibco, United States) containing 10% FBS, penicillin (100 U/ml), and streptomycin (100 μg/ml) at 37°C in a humidified atmosphere containing 5% CO_2_. AZT (Sigma-Aldrich, United States) was dissolved in dimethylsulfoxide (DMSO; SolarBio, Beijing, China) at 10 mg/ml and stored at −80°C. AZT was diluted with Dulbecco’s Phosphate-Buffered Saline (D-PBS) before use to ensure that the DMSO concentration was less than 0.5% (v/v).

### CCK-8 Assay to Determine Cell Viability at Various Zidovudine Concentrations

In 96-well microplates (Corning, United States), DF-1 cells were inoculated at a seeding density of 5 × 10^3^ cells per well in 100 μl of DMEM. Then, DF-1 cells were cultured with different concentrations of AZT (0, 0.1, 0.5, 1, 2, 5, and 10 μg/ml) for 48 h. The cytotoxicity of various concentrations of AZT was assayed by a CCK-8 Kit (NCM Biotech, Suzhou, China) following the manufacturer’s specifications. Briefly, after 48 h of incubation, the supernatant was removed and cells were washed three times with D-PBS and cultured with an equal volume of fresh medium. Then, 10 μl of CCK-8 solution was immediately added to each well and samples were cultured in a 5% CO_2_ incubator for 4 h. The absorbance value of 450 nm was detected by a microplate reader (Thermo Fisher Scientific, United States), and the cell viability (CV) was calculated according to Equation 1.


(1) $$\text{CV}=\frac{\text{A}\,\left(\mathrm{Drug}\right)-\text{A}\,\left(\mathrm{Blank}\right)}{\text{A}\,\left(\mathrm{Control}\right)-\text{A}\,\left(\mathrm{Blank}\right)}\times 100\%$$

### Dose-Response Curve to Analyze the Antiviral Effect of Zidovudine on Avian Leukosis Virus Subgroup J

Zidovudine treatment was carried out in 96-well microplates. DF-1 cells (5,000 cells per well) were infected with SDAU1005 stock (50 TCID_50_) in microplates prefilled with 100 μl of AZT with different concentrations (threefold serial dilutions). A column of DF-1 cells contains no drugs as positive control and a column of DF-1 cells contains no cells as blank control. The supernatant was harvested after the cells were cultured for an additional 5 days, and then, the ALV-p27 antigen was detected by an Avian Leukosis Virus Antigen Test Kit (IDEXX, United States) following the manufacturer’s specifications. The absorbance value of 650 nm was detected by a microplate reader, and the S/P value (samples OD value − negative control value)/(positive control value − negative control value) was calculated according to the formula in instructions. The inhibition rate of virus (IV) was calculated according to Equation 2 and represents the proportion of virus titer reduction under drugs.


(2) $$\text{IV}=\left(1-\frac{\text{S}/\text{P}\,\left(\mathrm{Drug}\right)-\text{S}/\text{P}\,\left(\mathrm{Blank}\right)}{\text{S}/\text{P}\,\left(\mathrm{Control}\right)-\text{S}/\text{P}\,\left(\mathrm{Blank}\right)}\right)\times 100\%$$

The AZT concentration required to inhibit SDAU1005 replication by 50% (IC_50_) was generated by fitting the inhibition curves with four parameters nonlinear regression model.

### Construction of Short Hairpin RNA Expression Plasmids

**FIGURE 1:**
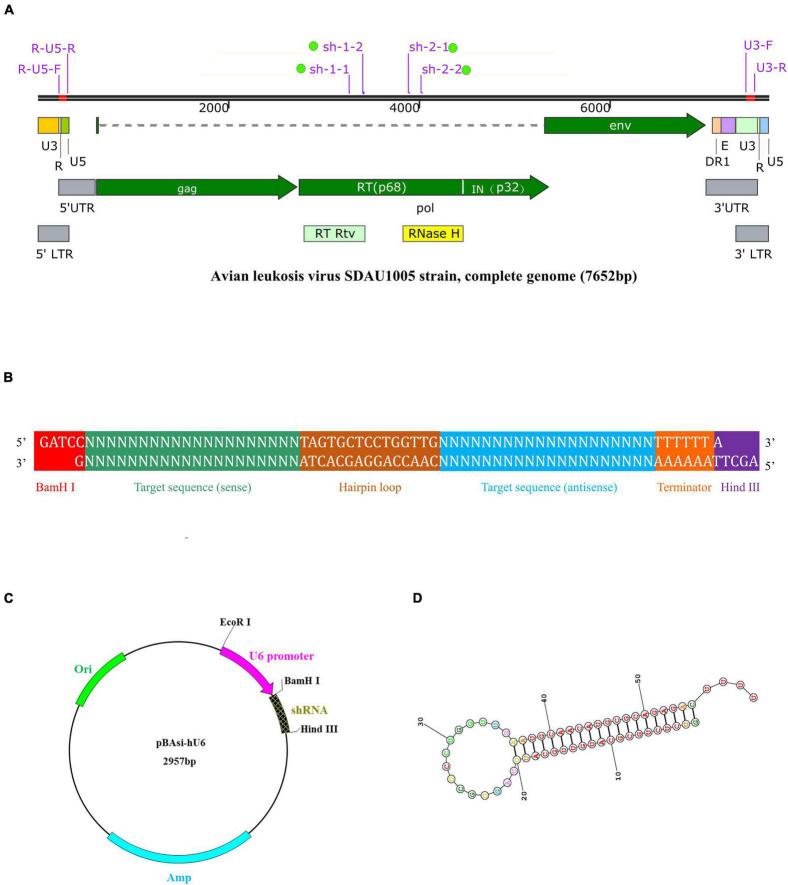
Schematic description of target ALV genomic features, shRNA expression cassette and vector, and shRNA structure. **(A)** Genomic structure of ALV-J stain SDAU1005 and position of target shRNA: sh-1-1, sh-1-2, sh-2-1, and sh-2-2. **(B)** An inverted repeat is cloned into the shRNA expression vector. The inserted DNA is designed in the following order: BamH I restriction endonuclease site, sense sequence, hairpin loop structure, antisense sequence, termination signal, and Hind III restriction endonuclease site. The forward sequence and reserve sequence are both 67-nt long. **(C)** Map of the pBAsi-Hu6 shRNA expression vector. The inverted repeat is inserted into the corresponding site (BamH I/Hind III) of the vector. **(D)** The secondary structure of the shRNA predicted by RNA structure (http://rna.urmc.rochester.edu/RNAstructure.html).

On the basis of the RT gene sequence of the ALV SDAU1005 strain, shRNAs were designed by BLOCK-iT RNAi Designer (Invitrogen, United States^1^). Two shRNAs were designed and synthesized in the RT active domain (RT-Rtv) and ribonuclease H active domain (RNase H), respectively (Figure 1A). The sequences of shRNAs were subjected to BLASTN^2^ to verify their specificity. In addition, a nonspecific shRNA was designed to serve as a negative control shRNA that did not target the known avian genome and the known avian leukemia virus genome.

The complementary oligonucleotide sequences (Table 1) of shRNA, including sense sequence, a hairpin loop structure, antisense sequence, termination signal, and BamH I and Hind III restriction endonuclease site (Figure 1B), were synthesized by Takara (Beijing, China). The annealed shRNA duplexes were cloned into the linearized shRNA expression vector pBAsi-hU6 (Figure 1C). The recombinant shRNA expression plasmids were extracted by an E.Z.N.A. Endo-Free Plasmid DNA Maxi Kit (Omega, United States) following the manufacturer’s specifications and confirmed by PCR and sequence analysis (Sangon Biotech, Shanghai, China). Positive plasmids with the correct sequencing results were named sh-1-1, sh-1-2, sh-2-1, sh-2-2, and sh-NC, respectively. In addition, when the recombinant shRNA expression plasmids were transfected into DF-1 cells, they were transcribed into shRNA (Figure 1D).

**TABLE 1:** Synthetic shRNA oligonucleotide sequences.

Name		Sequences
sh-1-1	Top strand	5′-GATCCGTCTCTGCGCATGTTGCATTTAGTGCTCCTGGTTGAATGCAACATGCGCAGAGACTTTTTTA-3′
	Bottom strand	5′-AGCTTAAAAAAGTCTCTGCGCATGTTGCATTCAACCAGGAGCACTAAATGCAACATGCGCAGAGACG-3′
sh-1-2	Top strand	5′-GATCCGGCCCGGAGTACAATATCTTTAGTGCTCCTGGTTGAAGATATTGTACTCCGGGCCTTTTTTA-3′
	Bottom strand	5′-AGCTTAAAAAAGGCCCGGAGTACAATATCTTCAACCAGGAGCACTAAAGATATTGTACTCCGGGCCG-3′
sh-2-1	Top strand	5′-GATCCGGCAAGGAGGTTGATATCCTTAGTGCTCCTGGTTGAGGATATCAACCTCCTTGCCTTTTTTA-3′
	Bottom strand	5′-AGCTTAAAAAAGGCAAGGAGGTTGATATCCTCAACCAGGAGCACTAAGGATATCAACCTCCTTGCCG-3′
sh-2-2	Top strand	5′-GATCCGGCGCGTCCACTGCATGTTTTAGTGCTCCTGGTTGAAACATGCAGTGGACGCGCCTTTTTTA-3′
	Bottom strand	5′-AGCTTAAAAAAGGCGCGTCCACTGCATGTTTCAACCAGGAGCACTAAAACATGCAGTGGACGCGCCG-3′
sh-NC	Top strand	5′-GATCCGTCTTAATCGCGTATAAGGCTAGTGCTCCTGGTTGGCCTTATACGCGATTAAGACTTTTTTA-3′
	Bottom strand	5′-AGCTTAAAAAAGTCTTAATCGCGTATAAGGCCAACCAGGAGCACTAGCCTTATACGCGATTAAGACG-3′

### Construction of Reverse Transcriptase-EGFP Reporter Plasmid

The sequence of RT gene was amplified from the infectious clone of ALV strain SDAU1005, and the EcoR I and Sal I restriction endonuclease sites were linked to both ends, respectively, of the RT gene using PCR amplification. The following pair of primers was used: EcoR-RT-F: 5′-GAATTCTATGACTGTTGCGCTACATCTGGCTAT-3′ and Sal-RT-R: 5′-GTCGACTTAATACGCTTGAAAGGTGGCTTGG-3′ for the amplification of RT gene of ALV-J. Then, the PCR products were purified using a Gel Extraction Kit (Omega, United States) and subcloned into a pMD18-T vector (Takara, Beijing, China). The recombinant plasmids (pMD-RT) were extracted using a Plasmid DNA Mini Kit (Omega, United States). As a next step, to construct the EGFP reporter plasmid, pMD-RT plasmid was digested with EcoR I and Sal I and cloned into the linearized pEGFP-C1 vector. The endotoxin-free plasmid (RT-EGFP) was extracted and confirmed using the above method.

### Co-transfection of Reverse Transcriptase-EGFP Reporter Plasmid and Short Hairpin RNA Expression Plasmids

For RT-EGFP reporter plasmid and shRNA expression plasmids co-transfection, DF-1 cells were inoculated in 12-well plates (Corning, United States) at a seeding density of 2 × 10^5^ cells per well and continued to culture in a CO_2_ incubator until the cells reached approximately 80% confluence. After that, 1.6 μg each of the shRNA expression plasmids (sh-1-1, sh-1-2, sh-2-1, sh-2-2, and sh-NC) was co-transfected with 1.6 μg RT-EGFP reporter plasmid to DF-1 cells using Lipofectamine™ 2000 (Invitrogen, United States) following the manufacturer’s specifications. Briefly, after 6 h of incubation with opti-MEM™ I Reduced Serum Medium (Gibco, United States), the supernatant was removed and cells were washed three times with D-PBS and cultured with an equal volume of fresh DMEM containing 10% FBS.

### Analysis of Reverse Transcriptase-EGFP Fusion Protein and Reverse Transcriptase mRNA Expression in DF-1 Cells

At 48 h after co-transfection, the supernatant was removed and DF-1 cells were washed thrice gently with D-PBS. DF-1 cells were fixed using 4% paraformaldehyde for 15 min and then incubated with 4,6-diamidino-2-phenylindole (DAPI; Sigma-Aldrich, United States) for 5 min before visualizing the expression of RT-EGFP fusion proteins using fluorescence microscopy (Nikon, 200×). At the same time, other DF-1 cells were washed thrice gently with D-PBS, trypsinized, and resuspended in cold D-PBS, and then, the percentage of EGFP fusion protein–positive cells was determined by LSRFortessa flow cytometer (BD, United States).

At 48 h after co-transfection, another group was harvested. Real-time PCR was later used to analyze the effect of shRNA interference on RT gene expression. Briefly, total RNA was extracted by an E.Z.N.A. Total RNA Kit (OMEGA, United States) following the manufacturer’s specifications, and then, RNA concentrations were quantified using a NanoDrop ND-1000 spectrophotometer (Thermo Fisher Scientific, United States). Reverse transcription and genomic DNA removal were performed using a PrimeScript RT reagent Kit with gDNA Eraser (Takara, Beijing, China), and about 1 μg of total RNA was reverse-transcribed into cDNA. Real-time PCR were performed on a Light Cycler 96 (Roche, Switzerland) using TB Green Premix Ex Taq (Takara, Beijing, China) following the manufacturer’s specifications, and two pairs of primers (RT-F/R and *ACTB*-F/R, Table 2) were used to amplify 2 μl of cDNA template in 20-μl reaction system. The real-time PCR thermal cycling conditions were 95°C for 5 min, followed by 40 cycles of denaturation at 95°C for 5 s and annealing and extension at 60°C for 34 s. The expression levels of the RT gene were normalized to the *β-actin* gene (*ACTB*, housekeeping gene), and the analyses of RT mRNA relative expression in each sample were performed by the 2^–ΔΔCT^ method.

**TABLE 2:** Real-time PCR primers used in this study.

Name	Sequences
RT-F	5′-CTAACGAGGCGAGGGAATG-3′
RT-R	5′-TTGGTGGGTTGGGTGGAGA-3′
ALV-F	5′-CAGAGAAGATACGGGTGGAAG-3′
ALV-R	5′-CTATGACAAGCAATGCAAACAG-3′
*ACTB*-F	5′-GAGAAATTGTGCGTGACATCA-3′
*ACTB*-R	5′-CCTGAACCTCTCATTGCCA-3′
R-U5-F	5′-GCCATTTTACCTCCCACCACA-3′
R-U5-R	5′-GCAGGTGTTCGTAATCGTCAGG-3′
U3-F	5′-GTCATGGTGTGATCGTGCC-3′
U3-R	5′-TCTCTCTGCAACGCGGAAC-3′
*HMG-14b-F*	5′-ACTGAAGAGACAAACCAAGAGC-3′
*HMG-14b-R*	5′-CCAGCTGTTTTAGACCAAAGAATAC-3′

### CCK-8 Assay to Determine the Cell Viability of Short Hairpin RNA Combined With Zidovudine

In 96-well microplates, AZT (1 μg/ml) was incubated with DF-1 cells transfected with 0.16 μg of different shRNA expression plasmids, respectively, and CV was detected and calculated as previously described.

### Virus Infection and Combination Therapy

First, in 12-well plates, DF-1 cells were inoculated at a seeding density of 2 × 10^5^ cells per well and continued to culture until the cells reached approximately 80% confluence. Then, the shRNA expression plasmids were transfected using Lipofectamine 2000 so that they were transcribed intracellularly and produced RNAi effects. Next, 8 h after transfection, the supernatant was removed, and cells were washed three times with D-PBS and cultured with an equal volume of fresh medium containing AZT (1 μg/ml) so that both shRNA targeting RT gene and AZT play an antiviral role in DF-1 cells. ALV-J strain SDAU1005 stock (300 TCID_50_), meanwhile, was inoculated into each well of 12-well plates. Culture supernatant and DF-1 cells were harvested at 24/48 h post-infection (hpi).

### Quantification of Viral RNA Loads

Viral RNA loads in DF-1 cells were quantified by the real-time PCR method (primers ALV-F and ALV-R, Table 2) as previously described, and the levels of ALV RNA load were normalized to the *β-actin* gene (primers *ACTB*-F and *ACTB*-R, Table 2). In addition, to determine the viral loads in cell culture supernatant at 48 hpi, an absolute quantitative real-time PCR of ALV-J was established. Put simply, the fragment amplified by primer ALV-F/R was cloned into a pMD18-T vector to construct the standard plasmid pMD-ALV, and the plasmid was then determined using a spectrophotometer and diluted 10-fold series with Tris-EDTA buffer (pH = 8.0) to construct a standard curve. Viral RNA was extracted by an E.Z.N.A. Total RNA Kit (OMEGA, United States) following the manufacturer’s specifications, determined using a spectrophotometer, and reverse-transcribed into cDNA. ALV-J loads in the culture supernatant were detected by real-time PCR using cDNA templates, calculated according to the standard curve, and normalized to per 100 μl of culture supernatant.

### Quantification of Pro-viral DNA Loads

After DF-1 cells were harvested at 24 hpi, genomic DNA was then extracted using a DNeasy Blood & Tissue Kit (Qiagen, United States) following the manufacturer’s directions, followed by detection of the pro-viral DNA loads of ALV-J using real-time PCR. The primers used for real-time PCR are shown in Table 2. According to the previous methods (Julias et al., 2001), primers were designed to determine the ALV-J pro-viral DNA loads, in which primers R-U5-F/R were used to determine the synthesis of minus-strand strong stop DNA(-sssDNA) of ALV-J and primers U3-F/R are used to amplify the ALV-J DNA specific for U3 to determine the amount of first-strand transfer. The pro-viral DNA loads (-sssDNA/U3DNA) of ALV-J were normalized to the *HMG-14b* gene [single-copy gene in the chicken genome (Srikantha et al., 1990)], and the analyses of their expression levels in each sample were performed by the 2^–ΔΔCT^ method.

### Western Blot to Analyze the Expression of Viral Protein in DF-1 Cells

DF-1 cells were harvested at 48 hpi and lysed on ice for 10 min using a cell lysis buffer (Beyotime Biotechnology, Shanghai, China) following the manufacturer’s specifications. After centrifugation at 14,000 × *g* for 5 min at 4°C, the supernatant was collected and then assayed for total protein content using a BCA Protein Quantification Kit (TransGen Biotech, Beijing, China), and 20 μg of total protein was subjected to 8% SDS-PAGE and transferred to a PVDF membrane (Millipore, United States). PVDF membranes were blocked using 5% skim milk for 1 h at room temperature, incubated with mouse anti-ENV mAb JE9 (Qin et al., 2001) or rabbit anti-ACTB antibody (Abcam, United Kingdom) at 4°C overnight, washed with Tris-Buffered Saline with Tween 20 (TBST) for three times, then incubated with HRP-conjugated goat anti-rabbit antibody (Abcam, UK) or goat anti-mouse antibody (Abcam, UK) at room temperature for 2 h, and detected with SuperSignal West Pico PLUS chemiluminescence substrate after TBST cleaning for three times. Exposure development was performed using a chemiluminescence imaging system (Azure Biosystems, United States).

### Indirect Immunofluorescence Assay to Analyze the Expression of Viral Protein in DF-1 Cells

DF-1 cells were harvested at 48 hpi, washed thrice gently with D-PBS, fixed with 4% paraformaldehyde for 15 min, blocked with QuickBlock Blocking Buffer for Immunol Staining (Beyotime Biotechnology, Shanghai, China), incubated with mouse anti-ENV antibody (mAb JE9), washed thrice with Phosphate-Buffered Saline with Tween 20 (PBST), incubated with FITC-conjugated goat anti-mouse antibody (Abcam, United Kingdom), washed thrice with PBST again, and then incubated with DAPI for 5 min before visualizing the expression levels of viral protein in DF-1 cells using fluorescence microscopy (Nikon, 200×).

### ELISA to Analyze ALV-p27 Levels in the Supernatant of DF-1 Cells

The supernatant of DF-1 cells was harvested at 48 hpi, and then, the ALV-p27 antigen was detected by a Leukosis Virus Antigen Test Kit (IDEXX Laboratories Inc., United States) following the manufacturer’s specifications. The absorbance value of 650 nm was detected by a microplate reader, and the S/P value was calculated according to the formula in instructions.

### Dose-Response Curve to Analyze Antiviral Effect of Short Hairpin RNA Combined With Zidovudine

In 96-well microplates, DF-1 cells were transfected with 0.16 μg of different shRNA expression plasmids, infected with SDAU1005 stock after 8 h of transfection, and treated with various concentrations of AZT, according to the methods mentioned above. Similarly, according to the methods mentioned above, the inhibition rates of virus in each group were detected and calculated, and the IC_50_ values were generated by fitting the inhibition curves. For the combination of AZT with different shRNA expression plasmids, the fold change of IC_50_ (FCIC_50_) was calculated with Equation 3.


(3) $${\text{FCIC}}_{50}=\frac{{\text{IC}}_{50}\,\left[\text{Mock}\,\text{shRNA}\right]}{{\text{IC}}_{50}\,\left[\text{ALV}-\text{targeted}\,\text{shRNA}\right]}$$

### Statistical Analysis

The results were expressed as the means ±SDs, and statistical analysis was performed by one-way ANOVA in SPSS 25.0 (SPSS Inc., Chicago, IL, United States). According to Duncan’s multiple-range test, *P* < 0.05 was considered statistically significant.

## Results

### Effect of Different Concentrations of Zidovudine on Cell Viability

**FIGURE 2:**
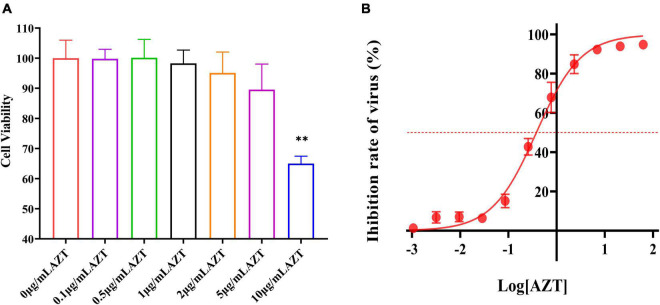
Antiviral effect and cell viability of AZT at different concentrations. **(A)** Cell viability of DF-1 cells after AZT treatment at 0.1, 0.5, 1, 2, 5, and 10 μg/ml for 48 h by CCK-8 assay. The experiment was repeated three times. The error bars represent the SD. ^**^P < 0.01 and ns represents not statistically significant. **(B)** Dose-response curve of AZT reveals the dose relationship between AZT and ALV-J. The *x*-axis represents the log of the concentration of AZT (μg/ml), and the *y*-axis represents the inhibition rate of virus (%), which was calculated according to Equation 2 and represents the proportion of virus titer reduction under drugs. The red dotted line indicates the position of 50% inhibition rate. The experiment was repeated eight times. The error bars represent the SD.

High concentrations of drugs are usually accompanied by cytotoxicity. In this study, the effect of AZT at different concentration on CV was determined by the CCK-8 assay. As shown in Figure 2A, AZT (10 μg/ml) induced a significant damage on DF-1 cells, but that of lower concentration is not significant. Briefly, CV was above 90% at 5 μg/ml; moreover, when using a drug concentration of 1 μg/ml, the CV was above 95%, suggesting that AZT at this concentration caused less damage to DF-1 cells.

### Antiviral Effect of Zidovudine at Different Concentrations

The inhibiting effect of AZT on ALV-J infection was further determined in this study, and the results were presented by a dose-response curve (Figure 2B). It was found that the antiviral effect is linear with the concentration from 0.01 to 10 μg/ml. Moreover, the IC_50_ of ALV-J strain SDAU1005 was 0.379 μg/ml. On the basis of the cytotoxicity of different concentrations of AZT determined in the previous section and the antiviral effect of different concentrations of AZT determined in this section, AZT at a concentration of 1 μg/ml (inhibit rate: >70%) was used in the next study to explore the effect of shRNA combined with AZT on ALV-J.

### Specific Short Hairpin RNAs Downregulated the Expression of Reverse Transcriptase in DF-1 Cells

**FIGURE 3:**
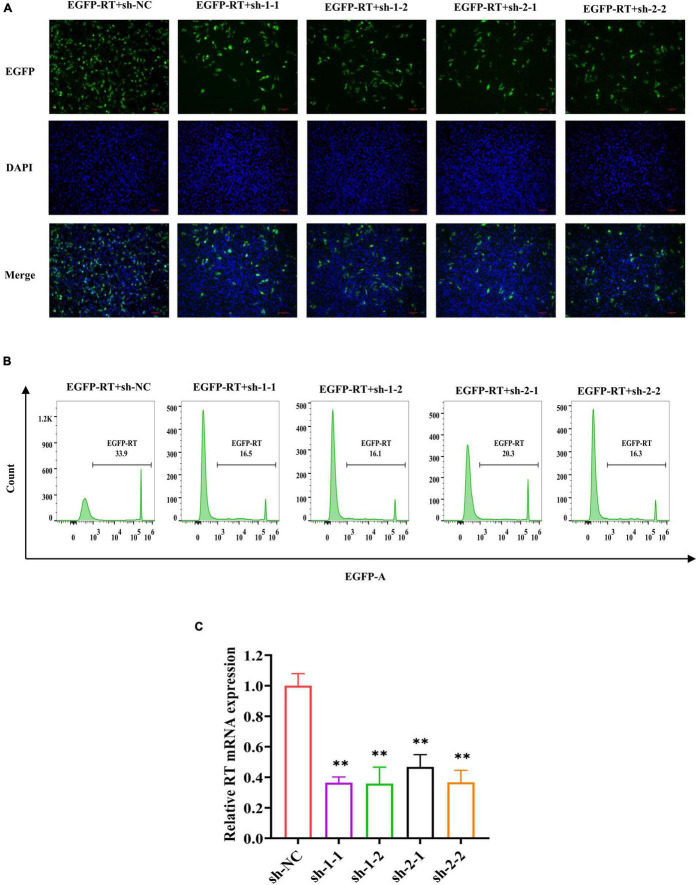
Specific shRNA inhibits RT RNA transcript levels and RT-EGFP fusion protein expression. **(A)** EGFP fluorescence in DF-1 cells co-transfected with RT-EGFP fusion protein expression plasmid and shRNA expression plasmid were observed (magnification, ×200) at 48 h post-transfection. **(B)** The percentage of EGFP-positive cells was determined by a flow cytometer. The *x*-axis represents the fluorescence intensity of EGFP, and the *y*-axis represents the EGFP-positive cell count. The criterion of positive cells was that the fluorescence intensity of DF-1 cells was higher than 10^3^. **(C)** Quantitative analysis of relative RT mRNA expression in DF-1 cells. The RNA transcript levels were determined by real-time PCR and normalized to *β-actin*. The experiment was repeated three times. The error bars represent the SD. ^**^P < 0.01 and ns represents not statistically significant.

To evaluate whether shRNA can knockdown the expression of RT gene in DF-1 cells, RT-EGFP expression plasmid and four shRNA expression plasmids targeting the different regions of RT were co-transfected into DF-1 cells, whereas sh-NC expression plasmid served as control. At 48 h after co-transfection, EGFP-related green fluorescence was observed using a fluorescence microscope. Compared with the negative control, four shRNAs all significantly knockdown the expression of RT-EGFP protein (Figure 3A). Further analysis of the co-transfected cells by flow cytometry revealed that the percentage of positive cells in each shRNA treatment group (sh-1-1, 16.5%; sh-1-2, 16.1%; sh-2-1, 20.3%; sh-2-2, 16.3%) was significantly decreased compared with the sh-NC group (33.9%) (Figure 3B). After that, we determined the mRNA transcript levels of RT gene in different groups by real-time quantitative PCR and found that the relative expression levels of each shRNA group were all significantly decreased relative to the sh-NC group (*P* < 0.01, Figure 3C). Taking the above results together, we concluded that all four specific shRNA expression vectors constructed in present study could significantly silence the expression of the RT gene.

### The Combination of Zidovudine and Short Hairpin RNA Showed Low Cytotoxicity

**FIGURE 4:**
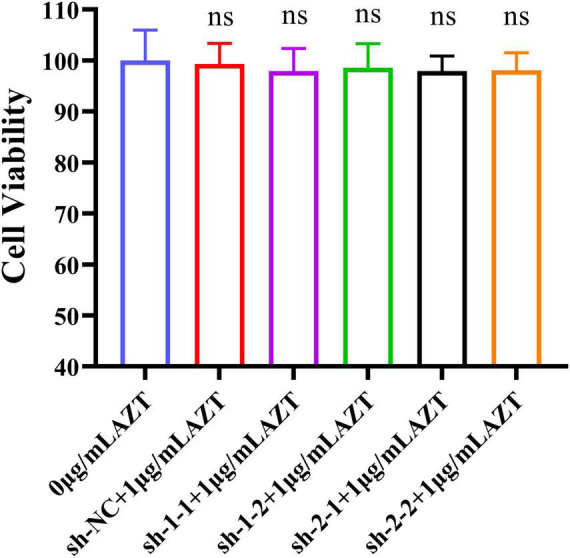
Cell viability of DF-1 cells after the co-treatment with AZT at 1 μg/ml and shRNA expression plasmid for 48 h by CCK-8 assay. The experiment was repeated three times. The error bars represent the SD. ns represents not statistically significant.

The previous works showed that AZT at a concentration of 1 μg/ml had less cytotoxicity (CV: >95%) and >70% antiviral rate, and the designed shRNAs could effectively suppress the expression of the RT gene. Furthermore, we determined the cytotoxicity of the combination of shRNA and AZT using CCK-8 assay. As the results shown in Figure 4, cell viabilities were above 95% when AZT and RNAi were used at the same time, indicating that this combination does not increase cytotoxicity.

### The Combination of Zidovudine and Short Hairpin RNA Could Significantly Inhibit the Replication of Avian Leukosis Virus Subgroup J in DF-1 Cells

**FIGURE 5:**
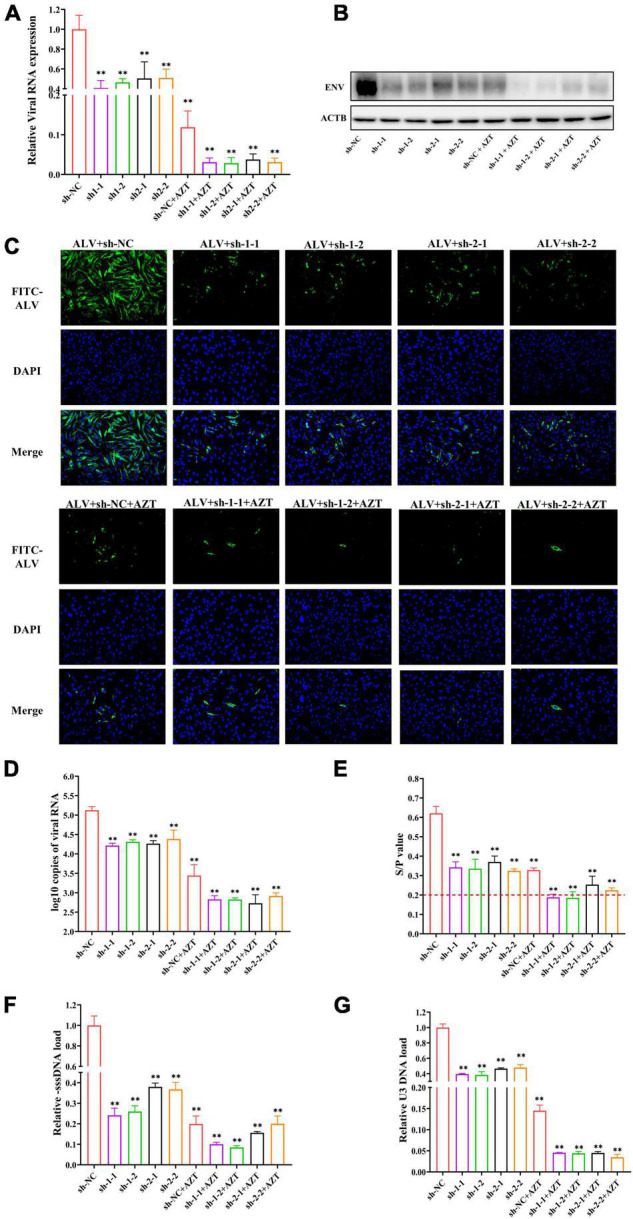
Additive antiviral activity of shRNA and AZT. **(A)** Quantitative analysis of relative ALV RNA expression in DF-1 cells. The viral RNA levels were determined by real-time PCR and normalized to *β-actin*. **(B)** Western blot analysis of ALV-J ENV and β-actin in DF-1 cells. The expression levels of the envelope protein of ALV-J were normalized against β-actin. **(C)** Immunofluorescence detection of ALV-J in DF-1 cells. The expression of envelope protein of ALV-J was observed under a fluorescence microscope (magnification, ×200). Bright green indicates the presence of ALV-J–positive cell, and blue represents nuclei. **(D)** ALV-J loads in culture supernatant. The copies of ALV-J RNA were detected by real-time PCR and normalized to per 100 μl of culture supernatant. **(E)** p27 antigen levels of ALV-J were detected by ELISA. The red dotted line indicates the cut-off value (S/P = 0.2) of the positive criteria. **(F,G)** Quantitative analysis of the synthesis or transfer of the minus strand DNA of ALV-J in DF-1 cells. The pro-viral DNA levels were determined by real-time PCR and normalized to *HMG-14b*. ^**^P < 0.01 and ns represents not statistically significant. All experiments were repeated three times. The error bars represent the SD.

We quantified the viral RNA loads in DF-1 cells of each group by real-time PCR to determine whether the combination of AZT and shRNA could significantly inhibit the replication of ALV-J in DF-1 cells. The group treated with sh-NC expression plasmid without AZT served as control. The results showed that, compared with the control group (Figure 5A), the relative viral load of ALV-J genomic RNA was significantly reduced under the combination of AZT and RNAi. We further verified the above results by determining the expression levels of the envelope protein (ENV) of ALV-J by Western blot and IFA, which showed the consistent results (Figures 5B,C).

### The Combination of Zidovudine and Short Hairpin RNA Could Significantly Inhibit the Viral Shedding

We collected cell supernatants from the different treatment groups at 48 hpi and determined the copies of virions in the cell supernatants using real-time PCR (Figure 5D) and found that, compared with the negative control group (10^5.126 ± 0.093^ copies/100 μl), using AZT (10^3.443 ± 0.279^ copies/100 μl) or shRNA expression plasmids (10^4.216 ± 0.057^ copies/100 μl, 10^4.313 ± 0.051^ copies/100 μl, 10^4.266 ± 0.076^copies/100 μl, and 10^4.384 ± 0.230^ copies/100 μl) alone could significantly suppress the viral copies in the DF-1 cell supernatants, but the combination of them was able to further decrease the ALV-J copies (10^2.830 ± 0.095^ copies/100 μl, 10^2.826 ± 0.042^ copies/100 μl, 10^2.729 ± 0.218^ copies/100 μl, and 10^2.917 ± 0.079^ copies/100 μl) in the supernatants.

To further confirm that shRNA in combination with AZT could synergistically suppress the release of ALV-J, the levels of ALV-p27 antigen in the supernatants at 48 hpi were determined by ELISA. The results (Figure 5E) showed that, compared with the negative control group, both shRNA and AZT alone suppressed the replication of ALV-J, and DF-1 cells co-treated with AZT and specific shRNA exhibited a better effect of inhibiting ALV-J release. The S/P value of the co-treated groups (sh-1-1, 0.188 ± 0.0164; sh-1-2, 0.185 ± 0.0319; sh-2-1, 0.254 ± 0.0435; and sh-2-2, 0.224 ± 0.0128) were near the cut-off value of positive criteria of the p27 antigen test kit. In conclusion, according to the levels of p27 antigen in the culture supernatants, shRNA expression plasmids targeting RT gene designed in this study and AZT had synergistic role of inhibit the release of ALV-J.

### The Combination of Zidovudine and Short Hairpin RNA Could Significantly Inhibit the Pro-viral Loads of Avian Leukosis Virus Subgroup J in DF-1 Cells

In the process of ALV replication, the viral genomic RNA that enters the host cell is reverse-transcribed into a double-stranded DNA (pro-viral cDNA), and the formation of new ALV-J in the infected cell is the result of pro-viral DNA transcription and translation. Therefore, it is necessary to detect the pro-viral loads in DF-1 cells to explore the effect of combination therapy on the ALV-J pro-viral load in DF-1 cells. For this study, primers (location as shown in Figure 1A) were designed according to the method of Julias et al. (2001), and the HMG-14b gene was used as the housekeeping gene. The results showed that, compared with shRNAs targeting RNase H sequence (sh-2-1 and sh-2-2), shRNAs targeting RT-RTV sequence (sh-1-1 and sh-1-2) significantly reduced the -sssDNA load of ALV-J and further suppressed the synthesis of ALV-J minus strand DNA when treated in combination with AZT (Figure 5F). However, the combination of shRNA targeting RNase H sequence (sh-2-1 or sh-2-2) and AZT has little effect on the synthesis of ALV-J minus strand DNA compared with AZT alone (Figure 5F). The relative U3 DNA loads of DF-1 cells treated with shRNA expression plasmids, AZT, or their combination were shown in Figure 5G, from which we found that both shRNAs targeting RT-RTV sequence and shRNAs targeting RNase H sequence significantly reduce the U3DNA load of ALV-J and further suppress the transfer of ALV-J minus strand DNA when treated in combination with AZT.

### The Impact of Zidovudine in the Presence of Specific Short Hairpin RNA Pressure

**FIGURE 6:**
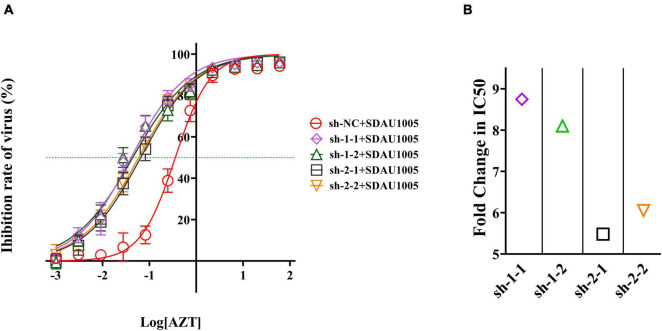
Antiviral activity of AZT was changed under the pressure of specific shRNA. **(A)** Dose-response curve of AZT in DF-1 cells expressing shRNAs. The *x*-axis represents the log of the concentration of AZT (μg/ml), and the *y*-axis represents the inhibition rate of virus (%), which was calculated according to Equation 2 and represents the proportion of virus titer reduction under drugs. The red dotted line indicates the position of 50% inhibition rate, according to which we can observe the shift of IC_50_. The experiment was repeated four times. The error bars represent the SD. **(B)** The fold change of IC_50_. The IC_50_ values were generated by fitting the inhibition curves, and the FCIC_50_ was calculated as Equation 3.

In previous studies, we found that the combination of shRNA and AZT might exhibit a synergistic or additive effects. To further confirm this, we explored the impact of AZT in the presence of shRNA pressure. Simply, supernatants were collected at 5 days post-infection, and the inhibitory activity of the antiviral drugs was calculated by comparing the p27 antigen levels of each group, after which dose-response curves were obtained and the IC_50_ values of ALV-J in different shRNA-transfected cells were generated by fitting the inhibition curves. The results were shown in Figure 6A, the inhibitory activity of drugs against viruses was changed under the combined effect of specific shRNA and AZT, and compared with the negative control group (sh-NC), dose-response curves for cells expressing specific shRNA (sh-1-1, sh-1-2, sh-2-1, or sh-2-2) shifted to the left, indicating that specific shRNAs can significantly increase the inhibitory activity of AZT on ALV-J. After that, we plotted the FCIC_50_ values of different specific shRNA transfection groups compared with sh-NC transfection group to further reveal the synergistic effect of shRNA and AZT in inhibiting ALV-J replication (Figure 6B), and the results showed that the combination of specific shRNA and AZT significantly increased the antiviral activity of AZT, and shRNAs targeting the RT-RTV sequence (sh-1-1 and sh-1-2) showed better synergistic activity than shRNAs targeting the RNase H sequence (sh-2-1 and sh-2-2).

## Discussion

Avian leukosis virus subgroup J infections have been reported worldwide and continue to pose a significant threat to the poultry industry (Landman et al., 2002; Fenton et al., 2005; Gao et al., 2010; Payne and Nair, 2012; Shittu et al., 2019). In addition, the mixed infection of ALV with other viruses or the contamination of it in live vaccines makes this disease more severe. However, up to now, there is no effective method to inhibit ALV virus replication *in vitro*; thus, it is difficult to de-contaminate the vaccine seed virus or purify the wild strains with the co-infection of ALV.

Although AZT and other RT inhibitors have a good inhibitory effect on retroviruses, it cannot be ignored that they also cause significant damage to host cells especially at a high concentration. Another noteworthy problem is that the antiviral mutants may be selected under the pressure of AZT, which leads to drug resistance (Meng, 2018). The application of multi-antiviral methods with different mechanisms to inhibit virus replication, often refer to as combination therapy, has always been a promising therapeutic (Adamson and Freed, 2010). It cannot only effectively inhibit virus replication but avoid generating drug-resistant strains (Hashmat et al., 2020). Therefore, we hope to use combinatorial antiviral therapy to inhibit the replication of ALV-J *in vitro* culture system, and our first thought is the combination of RNAi (Chen et al., 2004; Kanda et al., 2007; Gao et al., 2008; Wang et al., 2017) and AZT.

At present, there are several reports on the application of RNAi to inhibit ALV (Chen et al., 2007; Meng et al., 2011; Wei et al., 2015). Similarly, in this study, shRNA expression plasmids were used to inhibit the replication of ALV-J. Both ALV and HIV are retroviruses with similar genomic structure. The pol gene of ALV encodes RT (P68) and integrase (In, P32). RT includes RNA-dependent DNA polymerase active domain and RNase H active domain, which are necessary to complete viral reverse transcription (Herschhorn and Hizi, 2010; Justice and Beemon, 2013; Andrake and Skalka, 2015). Because RT is essential during ALV replication, shRNA expression plasmids targeting the key domains of ALV-J RT was designed at present study, and the successful application of shRNAs in inhibiting ALV-J RT gene expression in DF-1 cells was confirmed by co-transfection of shRNA expression plasmids and pEGFP-RT fusion protein expression plasmid.

After evaluating the cytotoxicity and antiviral activity of different concentrations of AZT *in vitro* culture system, we first determined that AZT at a concentration of 1 μg/ml was used in present study to explore the antiviral activity of the combination therapy, and then, we compared the antiviral effects of AZT and shRNA expression plasmids on ALV-J alone or in combination. We determined the relative expression of viral RNA and viral envelope protein in DF-1 cells and found that shRNA and AZT had a significant synergistic effect on inhibiting ALV-J replication in DF-1 cells. After that viral RNA copies and p27 antigen concentrations of ALV-J in culture supernatants were further detected by real-time PCR and ELISA. Similarly, the synergistic effect of the two was observed. We speculate that the reduction of ALV-J virions in the cell supernatant may be related to the inhibition of intracellular virus transcription or translation by AZT or shRNA, rather than both directly affecting virus release. In addition, we determined the synthesis and transfer of minus strand DNA during reverse transcription by detecting the relative expression of -sssDNA and U3 DNA of ALV-J, to explore the effect of the combination of shRNA and AZT on ALV-J provirus load in DF-1 cells. It should be noted that, compared with shRNAs targeting RNase H active domain (sh-2-1 and sh-2-2), shRNAs targeting RT active domain (sh-1-1 and sh-1-2) significantly reduced the -sssDNA load of ALV-J and further suppressed the synthesis of ALV-J minus strand DNA when treated in combination with AZT (Figure 5F). We speculate that the reduction of ALV-J pro-viral loads may be due to the co-treatment of shRNA and AZT that further suppressed the RNA-dependent DNA polymerase activity of ALV-J and then affects the synthesis of viral negative strand DNA. After that, we further confirmed the synergistic effect of shRNA and AZT in inhibiting ALV-J replication by plotting the dose-response curve and calculating FCIC_50_ (Figure 6). The results showed that the specific shRNAs significantly increased the antiviral activity of AZT on ALV-J, and shRNAs targeting RT active domain showed better synergistic effect than those targeting the RNase H active domain. Overall, shRNA and AZT had a good effect on the inhibition of ALV-J, but shRNA therapy alone was less effective than AZT alone, and it would cause a significant damage to host cells if we continue to carry on a high dose of AZT. In the case of the co-treatment of shRNA and AZT, the inhibition rate of ALV-J was improved without increasing cytotoxicity of AZT.

Finally, it must be emphasized that shRNA or AZT cannot completely kill the virus. Although the combination of shRNA and AZT may effectively avoid the generation of drug-resistant strains (Presloid and Novella, 2015), drug-resistant mutations or shRNA target sequence mutations are still noteworthy (Westerhout et al., 2005; Berkhout and Das, 2012). In addition, the application scenario of this method may be limited. Firstly, it can be used to remove ALV from some contaminated vaccine strains. The transmission of ALV caused by vaccine contamination is emerging all over the world, and there is no clear plan on how to effectively decontaminate the ALV in contaminated vaccine strains (Mao et al., 2020). Our antiviral strategy can continuously inhibit ALV *in vitro passages*, thus increasing the possibility of obtaining clean vaccine seed virus through plaque screening. In addition, this method can be used to purify some wild viral strains mixed with ALV, especially the co-infection of ALV and other virus is very common (Su et al., 2019).

## Conclusion

In conclusion, shRNA in combination with AZT was able to significantly inhibit ALV-J replication *in vitro* culture system, which provides a reference basis for the development of effective method for suppressing the replication of ALV-J and other similar retroviruses.

## Data Availability Statement

The original contributions presented in the study are included in the article/supplementary material, further inquiries can be directed to the corresponding author.

## Author Contributions

QW and QS conceived and performed the experiments, analyzed the data, and drafted the manuscript. PZ supervised the project and revised the manuscript. YW, QW, BL, YL, YXL, RX, WS, and SC performed part of the experiments. All authors contributed to the article and approved the submitted version.

## Conflict of Interest

The authors declare that the research was conducted in the absence of any commercial or financial relationships that could be construed as a potential conflict of interest.

## Publisher’s Note

All claims expressed in this article are solely those of the authors and do not necessarily represent those of their affiliated organizations, or those of the publisher, the editors and the reviewers. Any product that may be evaluated in this article, or claim that may be made by its manufacturer, is not guaranteed or endorsed by the publisher.

## Footnotes

1
https://rnaidesigner.thermofisher.com

2
http://blast.ncbi.nlm.nih.gov/Blast.cgi
